# Ceramide induces pyroptosis through TXNIP/NLRP3/GSDMD pathway in HUVECs

**DOI:** 10.1186/s12860-022-00459-w

**Published:** 2022-12-14

**Authors:** Fangfang Liu, Yangyang Zhang, Yining Shi, Kai Xiong, Fugui Wang, Jin Yang

**Affiliations:** 1 Department of Respiratory and Critical Care Medicine, Second Affiliated Hospital of Anhui Medical University, Hefei, 230601 Anhui China; 2 Department of Respiratory and Critical Care Medicine, Chest Hospital of Anhui Province, Hefei, Anhui China; 3 Department of Emergency Medicine, Second Affiliated Hospital of Anhui Medical University, Hefei, Anhui China; 4 Institute of Respiratory Diseases, Second Affiliated Hospital of Anhui Medical University, Hefei, 230601 Anhui China

**Keywords:** Ceramide, Endothelial cell, Cell pyroptosis, TXNIP/NLRP3/GSDMD signalling pathway, Vascular endothelial cell

## Abstract

**Background:**

Pyroptosis of endothelial cells is a new cause of endothelial dysfunction in multiple diseases. Ceramide acts as a potential bioactive mediator of inflammation and increases vascular endothelial permeability in many diseases, whether it can aggravate vascular endothelial injury by inducing cell pyroptosis remains unknown. This study was established to explore the effects of C8-ceramide (C8-Cer) on human umbilical vein vascular endothelial cells (HUVECs) and its possible underlying mechanism.

**Methods:**

HUVECs were exposed to various concentrations of C8-Cer for 12 h, 24 h, 48 h. The cell survival rate was measured using the cell counting kit-8 assay. Western blotting and Real-time polymerase chain reaction (RT-PCR) were used to detect the pyroptosis-releated protein and mRNA expressions, respectively. Caspase-1 activity assay was used to detect caspase-1 activity. Hoechst 33342/propidium iodide double staining and flow cytometry were adopted to measure positive staining of cells. Lactate dehydrogenase release assay and enzyme-linked immunosorbent assay were adopted to measure leakage of cellular contents. FITC method was used to detect the permeability of endothelial cells. ROS fluorescence intensity were detected by flow cytometry.

**Results:**

The viability of HUVECs decreased gradually with the increase in ceramide concentration and time. Ceramide upregulated the expression of thioredoxin interacting protein (TXNIP), NLRP3, GSDMD, GSDMD-NT, caspase-1 and Casp1 p20 at the protein and mRNA level in a dose-dependent manner. It also enhanced the PI uptake in HUVECs and upregulated caspase-1 activity. Moreover, it promoted the release of lactate dehydrogenase, interleukin-1β, and interleukin-18. Meanwhile, we found that ceramide led to increased vascular permeability. The inhibitor of NLRP3 inflammasome assembly, MCC950, was able to disrupt the aforementioned positive loop, thus alleviating vascular endothelial cell damage. Interestingly, inhibition of TXNIP either chemically using verapamil or genetically using small interfering RNA (siRNA) can effectively inhibit ceramide-induced pyroptosis and improved cell permeability. In addition, ceramide stimulated reactive oxygen species (ROS) generation. The pretreatment of antioxidant N-acetylcysteine (NAC), ROS scavenger, blocked the expression of pyroptosis markers induced by C8-cer in HUVECs.

**Conclusion:**

The current study demonstrated that C8-Cer could aggravate vascular endothelial cell damage and increased cell permeability by inducing cell pyroptosis. The results documented that the ROS-dependent TXNIP/NLRP3/GSDMD signalling pathway plays an essential role in the ceramide-induced pyroptosis in HUVECs.

**Supplementary Information:**

The online version contains supplementary material available at 10.1186/s12860-022-00459-w.

## Introduction

Increased vascular endothelial injury and barrier dysfunction are hallmarks of many diseases, such as acute lung injury, cardiac arrest and acute myocardial infarction [1–3]. In addition to apoptosis and necrosis of endothelial cells under various inflammatory stimuli, numerous studies have shown that pyroptosis was involved in the vascular dysfunction [4–6]. Although the underlying molecular mechanisms of vascular dysfunction are not yet fully understood, a large body of studies showed that an increase in circulating ceramide concentration is one of the key factors [7]. Endothelial cells are widely used to study the role of ceramide in vascular function and related diseases, ceramide can be produced in endothelial cells through a variety of pathways as part of the cellular response to stress stimuli. Furthermore, ceramide is involved in the endothelial cell barrier breakdown and increased permeability [8, 9]. It is the main regulator of cell death by mainly promoting cell cycle arrest and apoptosis [10, 11]. However, it is unclear whether ceramide causes endothelial cell pyroptosis in sepsis, acute lung injury and others diseases.

The thioredoxin-interacting protein (TXNIP) is a negative regulator of the antioxidant thioredoxin [12]. Inhibition of thioredoxin by TXNIP promotes cellular oxidative stress and accelerates the inflammatory response [13, 14]. NLRP3 inflammasome can be activated after ROS-mediated interaction of TXNIP to NLRP3 in endoplasmic reticulum (ER) under stressed conditions [15]. The TXNIP-NLRP3 inflammasome is a macromolecular multi-protein compound consisting of TXNIP, NLRP3, adaptor protein ASC, and the effector protein caspase-1 [16]. TXNIP deficiency impairs the activation of the NLRP3 inflammasome and subsequent secretion of inflammatory cytokines, such as interleukin (IL)-1β [17, 18]. Our previous study has shown that the ASM/Cer/TXNIP signalling pathway is closely related to the activation of the inflammasome. While small interfering RNA (siRNA) and verapamil were used to inhibit TXNIP, we found that ceramide-induced the level of TXNIP expression and the activation of NLRP3 inflammasome was downregulated [19]. Nevertheless, further clarification is needed to elucidate the involvement of endothelial cell pyroptosis upon ceramide exposure and its possible relationship in the activation of the downstream pathway triggered by TXNIP.

Human umbilical vein endothelial cells (HUVECs) have been extensively used as models for the study of endothelial cell pyroptosis in sepsis and other diseases [5, 20, 21]. Cell pyroptosis is considered a programmed process of cell death associated with an inflammatory response, which is different from apoptosis. It leads to the formation of plasma membrane pores and the release of the inflammatory cytokines IL-1β and IL-18, and is characterised by the cleavage of its effector GSDMs induced by inflammatory caspases [22, 23]. Pyroptosis is closely associated with inflammasome activation. NLRP3 is a common type of inflammasome [24] that activates caspase-1. It leads to the maturation and secretion of IL-1β and IL-18, and the mediation of pyroptosis [25]. However, NLRP3-related pyroptosis in ceramide-induced endothelial cell injury is not yet well understood.

In the current study, we investigated the role of ceramide in promoting endothelial injury and explored the potential mediating role of the TXNIP /NLRP3/GSDMD pathway in pyroptosis.

## Methods

### Materials and antibodies

C8-ceramide was purchased from Cayman Chemical Company (Ann Arbor, MI, USA). Verapamil was obtained from Abmole Bioscience, Inc. (Texas, USA). The sodium salt CP-456773 (also known as MCC 950) was acquired from Sigma-Aldrich (St. Louis, MO, USA). Caspase-1 activity assay kit, Annexin V-FITC/PI apoptotic detection kit, and Hoechst 33342/PI kit were purchased from BestBio (Shanghai, China). Cell counting kit-8 (CCK-8) assay kit was purchased from Beyotime Biotechnology Company (Shanghai, China). Rabbit monoclonal antibodies against NLRP3 (no. ab263899), TXNIP (no. ab188865), GSDMD (no. ab210070), and cleaved N-terminal GSDMD (No. ab215203), caspase-1 (no. ab207802), β-actin (no. ab179467) were purchased from Abcam (Cambridge, UK).

### Cell culture and treatments

In our study, HUVECs purchased from ScienCell Research Laboratories and grown in extracellular matrix supplemented with 5% foetal bovine serum, 1% (volume per volume) penicillin/streptomycin, and 5% ECGS. The cells were then placed in a humidified incubator with 5% carbon dioxide at 37 °C. When the density of cells reached 65%, the cells were treated with ceramide (0, 5, 10, and 20 μM) for 12 h. In subsequent experiments, the HUVECs were randomly divided into different groups depending on treatment conditions.

### RNA silencing experiment

Small interfering RNAs (siRNAs) of TXNIP and negative control were synthesized by GenePharma (shanghai, china). After HUVECS of the 6-well plates had reached 40% confluency, Lipofectamine 2000™ (Invitrogen, Carlsbad, USA) was used to perform cell transfection. After TXNIP siRNA and HUVECS were co-incubated for 24 h, western blotting and quantitative real-time PCR were used to determine the transfection efficiency of si-TXNIP.

### Cell viability assays

When cells grew exponentially, the HUVECs were digested, centrifuged, and resuspended. They were then placed in 96-well plates (5 × 10^3^ cells/100 ul). After incubation for 24 h, the cells were then treated with various doses of ceramide for 12 h, 24 h, 48 h. Next, 10 μl of the CCK-8 reagent was added to each well to measure cell viability. The absorbance at 450 nm was measured using a Thermo Fisher microplate reader.

#### Determination of ROS

Reactive Oxygen Species Assay Kit (Beyotime, S0033) was used to detect ROS. After the drug treatment, the cells were incubated with DCFH-DA in a 37 °C incubator in the dark for 20 min.

### Western blotting

Total proteins were harvested from HUVECs with enhanced RIPA lysis buffer containing 1% phenylmethylsulfonyl fluoride (Beyotime, Shanghai, China) and phosphatase inhibitors (Beyotime, Shanghai, China). Cell lysates were collected and centrifuged at 13200 rpm and 4 °C for 30 min. Next, the BCA (Beyotime, Shanghai China) kit was used to determine the concentration of protein supernatant. Equal amounts of protein mixtures were separated by SDS-PAGE gel and transferred to polyvinylidene difluoride membranes (Millipore, USA). After the transfer, 5% skimmed milk was used to block non-specific binding sites on the membrane for 1 h at 37 °C. Subsequently, membranes were probed with the corresponding primary antibodies at 4 °C overnight. After thoroughly washing excess primary antibodies from the membrane with TBST, the membrane was transferred to the corresponding horseradish peroxidase conjugated secondary antibody for 2 h. Western blot bands were detected using an ultra-sensitive chemiluminescent substrate ECL luminescent solution and imaged using a Bio-Rad ChemiDoc TMMP system. Finally, the bands were analysed using ImageJ software tools.

### Quantitative real-time polymerase chain reaction

RNAiso Plus (Takara, Japan) was used to isolate total RNA from the drug-treated HUVECs. Homologous cDNA was synthesised using the PrimeScript™ RT Reagent Kit (Takara, Japan). Briefly, each enzyme-free EP tube contained 10 ul of a mixture of 5× PrimeScript RT Master Mix, extracted RNA, and RNase-free dH2O. The mixture was then used to perform reverse transcription reactions to synthesise cDNA. Next, cDNA, specific primers, sterile water, and TB Green Premix Ex Taq II (Takara, Japan) constituted the 25 ul PCR reaction system. PCR was performed using the CFX96 Real-Time PCR Detection System (Bio-Rad, USA). The two-ΔΔct method was used to calculate the relative expression of target genes.

### Caspase-1 activity assay

To understand caspase-1 activity in HUVECs treated with the drugs, we utilised caspase-1 activity assay kit to detect caspase-1 activity. Briefly, cells were harvested and lysed using 50 μl cold lysis buffer. The protein concentration was then determined using the BCA assay kit. Then, 50 μl of cleavage supernatant containing–100-200 μg protein, 50 μl of detection buffer, 10 μl of caspase-1 substrate were added to 96 -well plates. The absorbance was determined at 405 nm using a microplate reader.

### Hoechst 33342/PI staining

To evaluate cell death, the cells were treated with the drugs mentioned above, washed twice with ice-cold PBS, and were incubated with a mixed solution of 1 mL staining buffer, 10 ul Hoechst 33342, and 5 ul PI for 20 min at 4 °C in the dark. The stained cells were immediately captured using Olympus 1X71 fluorescent inverted microscope (Olympus, Jpan).

### Flow cytometry analysis

The pyroptotic cells were detected using the Annexin V-FITC/propidium iodide (PI) double staining assay. After drug treatment, the HUVECs were digested with trypsin without ethylene diamine tetraacetic acid (EDTA). The cells were then centrifuged at 500 rpm at 4 °C for 5 min to collect digested and floating cells. The acquired cell pellet was washed twice with ice-cold PBS and centrifuged again. After that, the cell pellet was re-suspended in 400 μl 1× Annexin V binding buffer at a concentration of 1 × 106/ ml and stained with 5 ul Annexin V-FITC and 7 ul PI staining solution for 20 min at 4 °C without light exposure. Finally, the treated cells were analysed using CytoFLEX flow cytometry.

### LDH release detection

After the cells were treated with the indicated drugs, the culture supernatants were collected for the subsequent experiments. Following the manufacturer’s instructions, the release of LDH was detected using an LDH assay kit (Jiancheng, Nanjing, China).

### Enzyme-linked immunosorbent assay

After the HUVECs reached 65% confluence, the cells were treated for the corresponding time in accordance with the above-mentioned experimental design, and ELISA kits (Elabscience, China) were used to measure the levels of inflammatory factors, including IL-18 and IL-1β, in cell culture supernatants according to the experimental instructions. IL-18 and IL-1β secretion levels in each group were normalised to the cell protein concentration of the corresponding group.

### Endothelial cell permeability assay

Transwell inserts with membrane pore diameter of 0.4um and growth area of 0.33cm^2^ were selected, and 70 ul of diluted matrix gel was added to the upper chambers. After the matrix gel has solidified, 3.5 × 10^^^4 HUVECS were seeded onto the transwell upper compartment and were grown to confluence. Then cells were treated with corresponding drug for 12 h, 200ul FITC-labeled dextran was added into the upper chamber and incubated at 37 °C for 1 h. Finally 100ul of the medium was collected from the upper and lower chamber, the fluorescence intensity was measured with a multifunctional microplate reader to determine the permeability of endothelial monolayer to dextran.

### Statistical analysis

Statistical analysis was performed using GraphPad Prism version 9.0 (GraphPad Software, USA). Differences among groups were estimated using one-way analysis of variance followed by Tukey’s post-hoc test. All of the data from three independent experiments are expressed as the mean ± standard deviation. The data are considered statistically significant when *P* values are < 0.05.

## Results

### Effects of C8-ceramide and verapamil on HUVECs’ viability

**Fig. 1:**
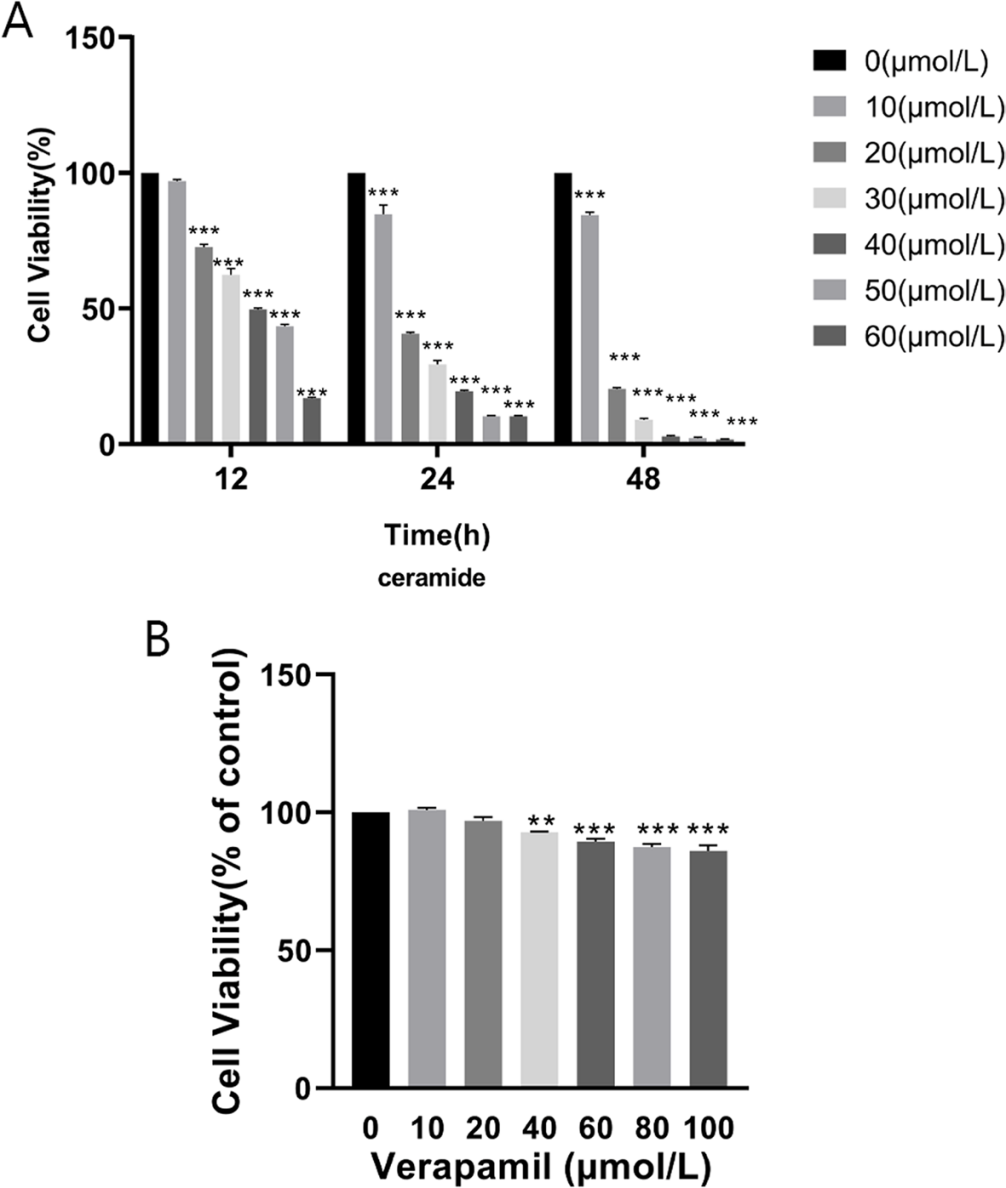
Effects of ceramide and verapamil on cell viability. Effects of ceramide (various concentrations and incubation times) on HUVECs cell viability in comparison to the corresponding control (**A**). HUVECs were treated with verapamil at a series of concentrations for 12 h (**B**). Values were reported (*n* = 3) as the mean ± standard deviation. ^*^P < 0.05, ^**^P < 0.01 and ^***^P < 0.001 vs. control

Different doses of C8-ceramide were applied to treat HUVECs for 12, 24 and 48 h. The cytotoxic effects of C8-ceramide (0, 10, 20, 30, 40, 50, 60 μM) on HUVECs were determined by CCK-8. As shown in Fig. 1A, the HUVECS survival rate decreased gradually with increasing ceramide concentration and time. Cell viability in the groups treated with 20 μM C8-ceramide for 12 h was approximately 70% of that in the control group. Based on the above experimental results, C8-ceramide (20 μM) for 12 h was selected for subsequent experiments. In view of this experiment, a 12 h incubation protocol was chosen to study the effects of different concentrations of verapamil on cell viability. HUVECs were exposed to 0, 10, 20, 40, 60, 80, and 100 μM verapamil for 12 h. The results indicated that no significant toxicity was observed in the 0 to 20 μM verapamil group (Fig. 1B). Hence, 20 μM verapamil was used in further experiments in this study.

### Changes of TXNIP expression and NLRP3 inflammasome activation by ceramide

**Fig. 2:**
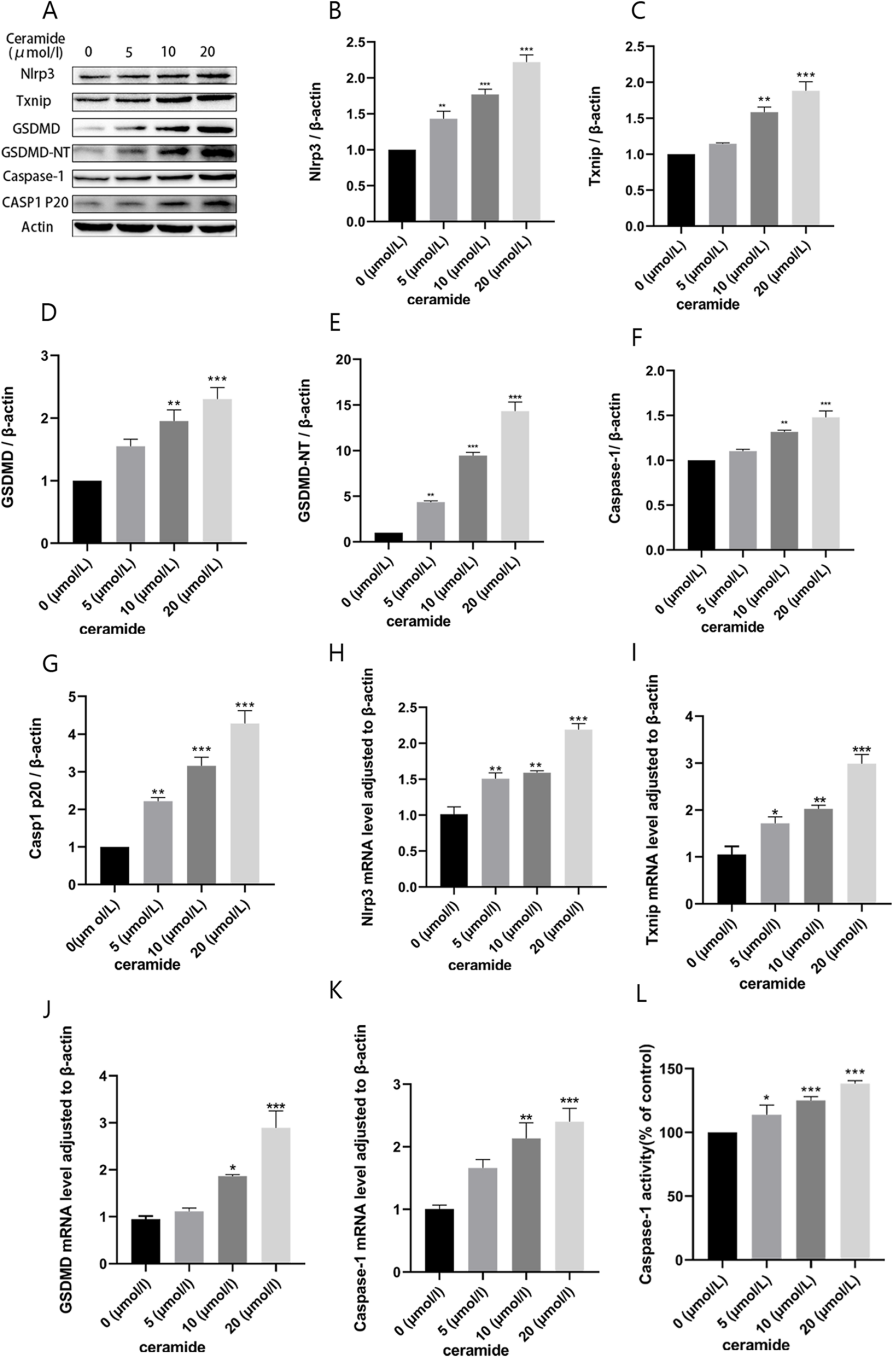
Ceramide promotes TXNIP expression and NLRP3 inflammasome activation. HUVECs were treated with 0, 5, 10, 20 μM ceramide for 12 h. Effects of ceramide on the protein expression: NLRP3 (**B**), TXNIP (**C**), GSDMD (**D**), GSDMD-NT (**E**), Caspase-1 (**F**), CASP1 P20 (**G**). Effects of ceramide on the mRNA expression: NLRP3 (**H**), TXNIP (**I**), GSDMD (**J**), Caspase-1 (**K**). Caspase-1 activity assay kit (**L**) was used to detected effect of ceramide on caspase-1 activity. Values were reported as the mean ± standard deviation in (**A–L**) (*n* = 3). The expression of β-actin was used as an internal control. ^*^P < 0.05, ^**^P < 0.01 and ^***^P < 0.001, compared with the control group

**Fig. 3:**
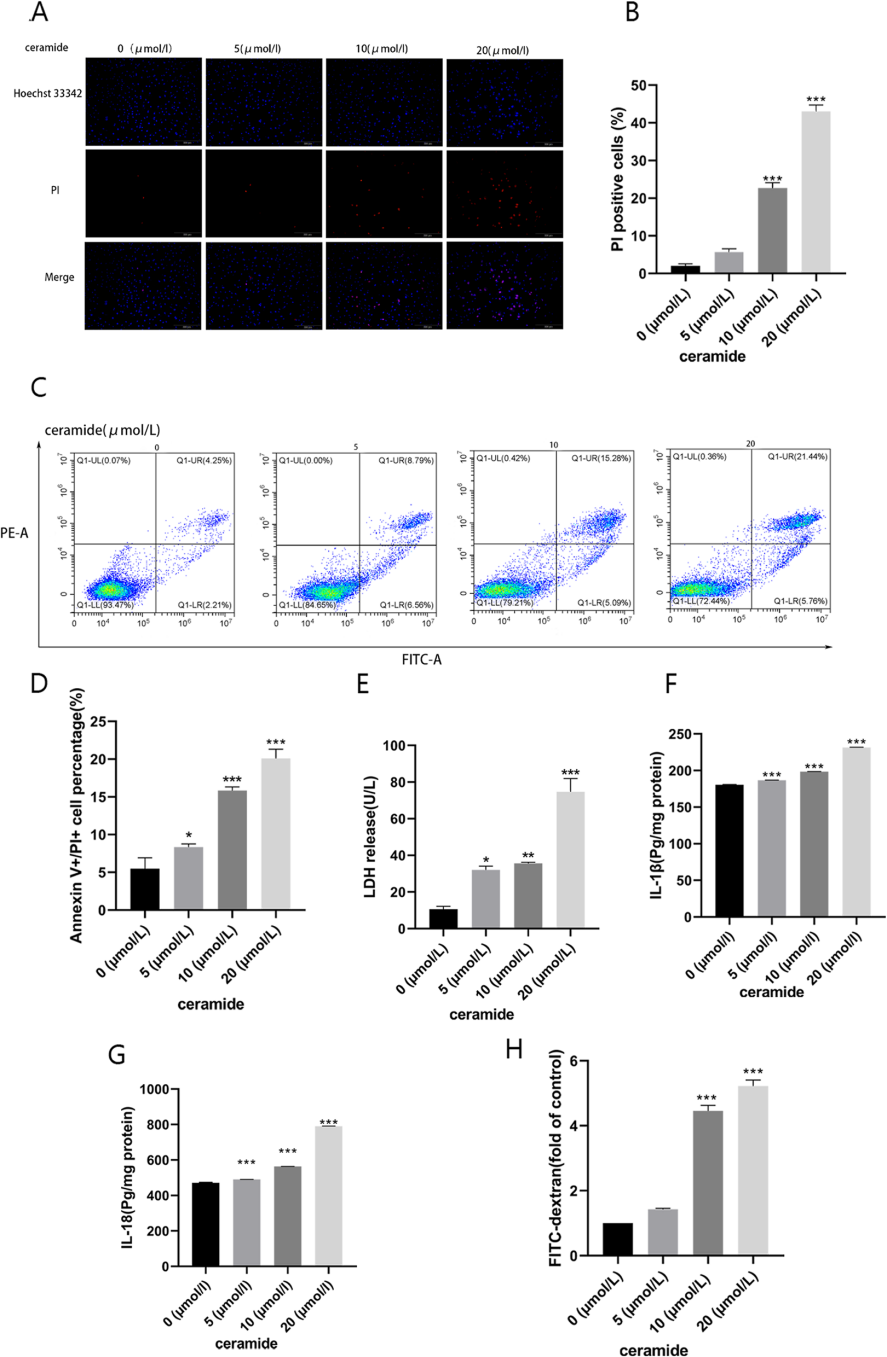
Dose response of the effects of ceramide on vascular endothelial injury. Hoechst33342/PI double staining (**A, B**) and Flow cytometry (**C, D**) were used to evaluate cell death. LDH kit (**E**) was used to detect cell LDH release. ELISA was used to analysed the level of IL-1β (**F**) and IL-18 (**G**) in the supernatant. FITC-dextran was used to evaluated cell permeability (**H**). Values were reported as the mean ± standard deviation in (**A–E**) (*n* = 3) and (**F-H**) (*n* = 5). ^*^P < 0.05, ^**^P < 0.01 and ^***^P < 0.001, compared with the control group. Images were captured from random fields of view at a magnification of × 100. Scale bar = 200 μM

We investigated NLRP3 inflammasome-dependent cell pyroptosis to explore the mechanism by which TXNIP facilitates HUVEC pyroptosis. Next, expression of TXNIP and the activation of NLRP3 inflammasome was determined, treated with 0, 5, 10, 20 μM ceramide for 12 h. As the ceramide concentration increased, the proteins levels of TXNIP, NLRP3, GSDMD, GSDMD-NT, caspase-1 and Casp1 p20 gradually increased (Fig. 2A-G). The mRNA expression level also exhibited similar results, as shown in Fig. 2H-K. At the same time, as previously mentioned, pyroptosis relies on the cleavage of GSDMD induced by activated caspase-1. GSDMD-NT results in the formation of membrane pores, thereby causing positive staining of cells and leakage of cellular contents. Consequently, we evaluated the activation of caspase-1, dead cell staining, and LDH release. The results showed that caspase-1 activity was enhanced (Fig. 2L). Furthermore, Hoechst 33342/PI staining (Fig. 3A-B) and flow cytometry (Fig. 3C-D) revealed that ceramide exposure increased PI uptake by HUVECs. The extracellular LDH content further verified the disruption of endothelial cell membrane. As expected, LDH release increased in the ceramide-treated group compared to that in the matched group (Fig. 3E). During pyroptosis, the release of pro-inflammatory cytokines, such as IL-1β and IL-18 can be released into the extracellular space. The ELISA results also verified the occurrence of this process (Fig. 3F-G). At the same time, FITC method was used to detect the permeability of endothelial cells in each treatment group, the result showed that ceramide treatment increased the permeability of HUVEC (Fig. 3H). These findings suggested that ceramide treatment promoted TXNIP expression and cellular NLRP3 inflammasome activation while led to vascular endothelial injury in HUVECs.

### Ceramide induced pyroptosis via NLRP3 inflammasome activation in HUVECs

**Fig. 4:**
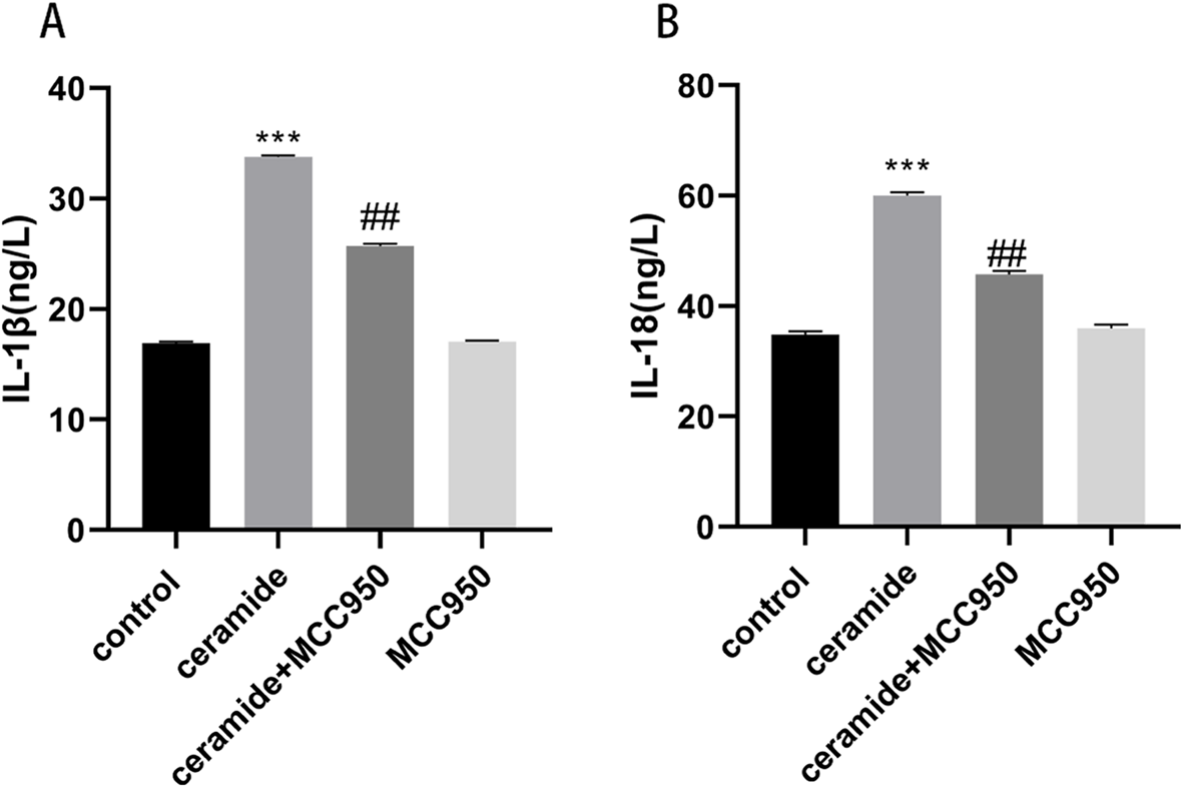
Ceramide-induced activation of the TXNIP/NLRP3/GSDMD pathway contributes to HUVECs’ pyroptosis. HUVECs were pre-treated with MCC950 (10uM) for 2 h followed by the addition of C8-ceramide(20 μM) for a further 12 hours. Effects of MCC950 on the expression of IL-1β (**A**) and IL-18 (**B**). Values were reported as the mean ± standard deviation in (**A–B**) (*n* = 3). ^*^P < 0.05, ^**^P < 0.01 and ^***^P < 0.001 vs control group. ^#^*P* < 0.05, ^##^*P* < 0.01 and ^###^*P* < 0.001 vs ceramide treatment group

To further illustrate the cause and effect, MCC950, a potent, selective, small-molecule inhibitor of NLRP3 inflammasome was used. Pre-treatment with MCC950 (10uM) markedly inhibited ceramide-induced expression of IL-1β and IL-18 in HUVECs (Fig. 4A-B). Based on all the above collective results, we postulated that C8-Cer induces pyroptosis through TXNIP/NLRP3/GSDMD pathway in HUVECs.

### Inhibition of TXNIP expression can reduce the ceramide-induced activation of subsequent pathways and improve vascular endothelial injury

**Fig. 5:**
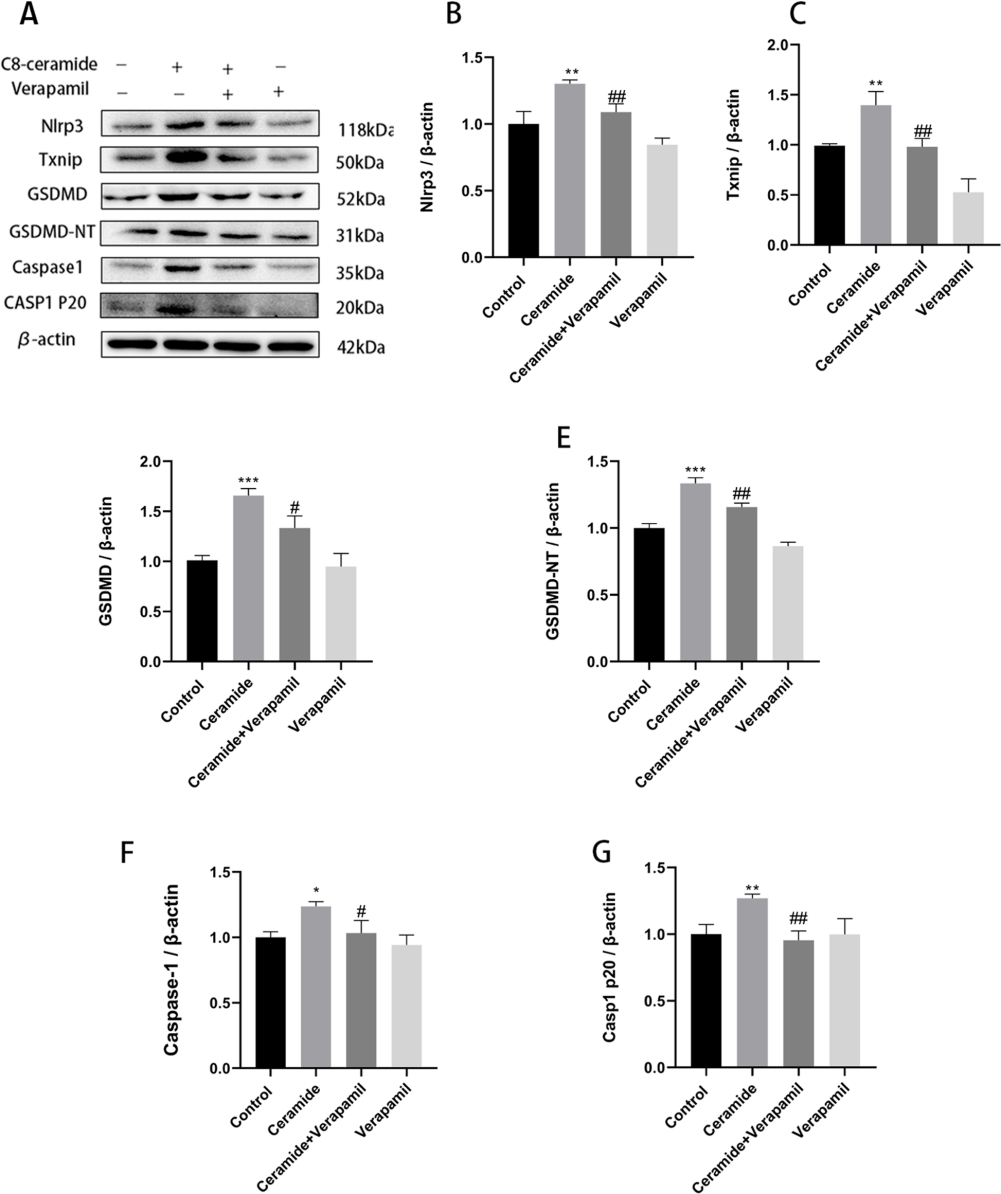
Verapamil: Inhibition of TXNIP attenuates ceramide-induced TXNIP expression and NLRP3 inflammasome activation. HUVECs were incubated with 20 μM verapamil for 2 h, followed by 20 μM ceramide for 12 h. Effects of verapamil on ceramide-induced pyroptosis-related proteins: NLRP3 (**B**); TXNIP (**C**); GSDMD (**D**); GSDMD-NT (**E**); Caspase-1 (**F**); Casp1 p20 (**G**). Values were reported as the mean ± standard deviation in (**A–G**) (*n* = 3). The expression of β-actin was used as an internal control. ^*^P < 0.05, **P < 0.01 and ***P < 0.001 vs control group. ^#^*P* < 0.05, ^##^*P* < 0.01 and ^###^*P* < 0.001 vs ceramide treatment group

**Fig. 6:**
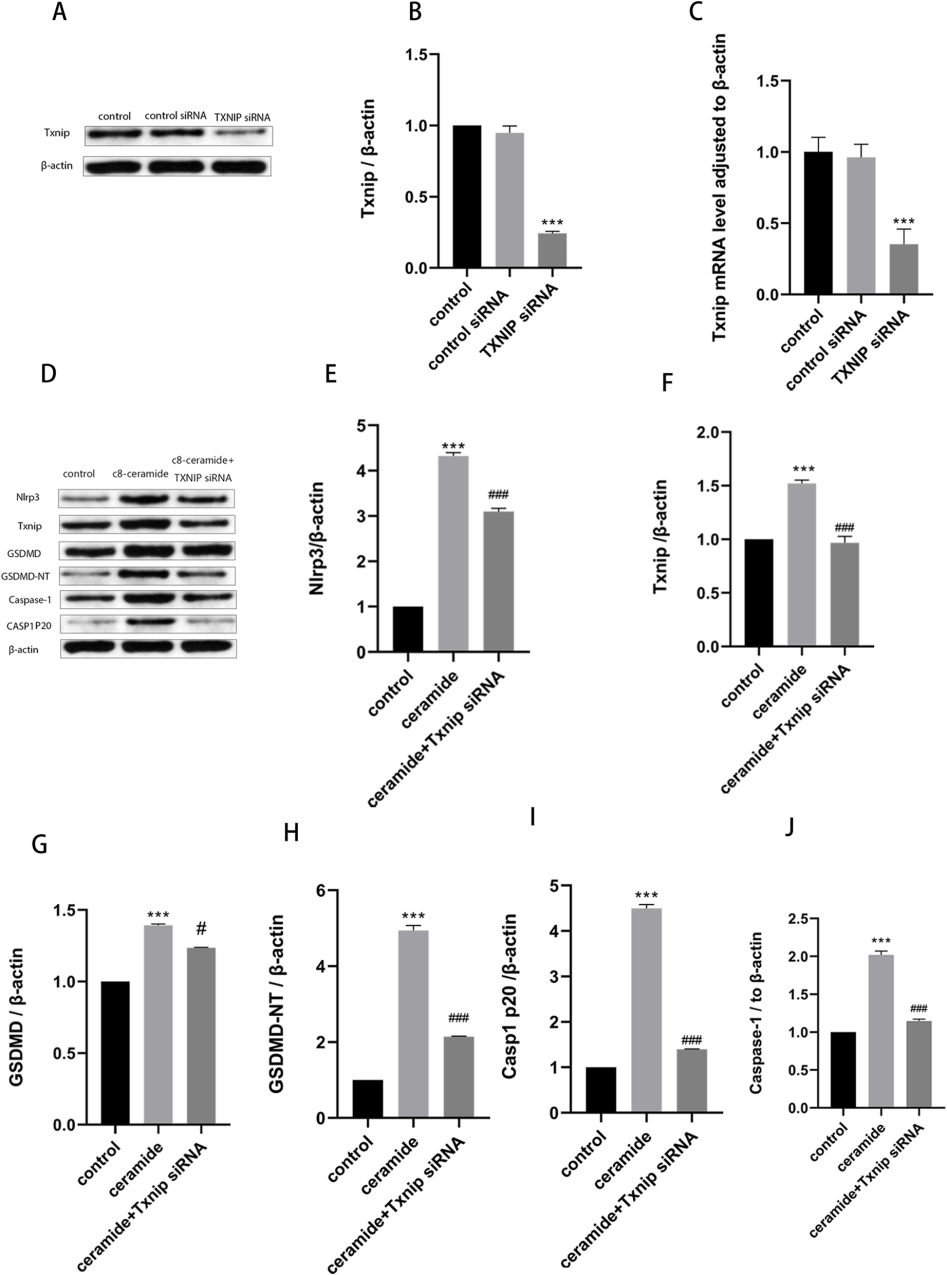
TXNIP siRNA effects ceramide-induced TXNIP expression and NLRP3 inflammasome activation. HUVECs were incubated with TXNIP siRNA for 24 h, followed by 20 μM ceramide for 12 h. Effects of small interfering RNA on the expression of TXNIP (**A-C**). Effects of si-TXNIP on ceramide-induced pyroptosis-related proteins: NLRP3 (**E**); TXNIP (**F**); GSDMD (**G**); GSDMD-NT (**H**); Caspase-1 (**I**); Casp1 p20 (**J**). Values were reported as the mean ± standard deviation in (**A–J**) (*n* = 3). The expression of β-actin was used as an internal control. *P < 0.05, **P < 0.01 and ***P < 0.001 vs control group. ^#^*P* < 0.05, ^##^*P* < 0.01 and ^###^*P* < 0.001 vs ceramide treatment group

**Fig. 7:**
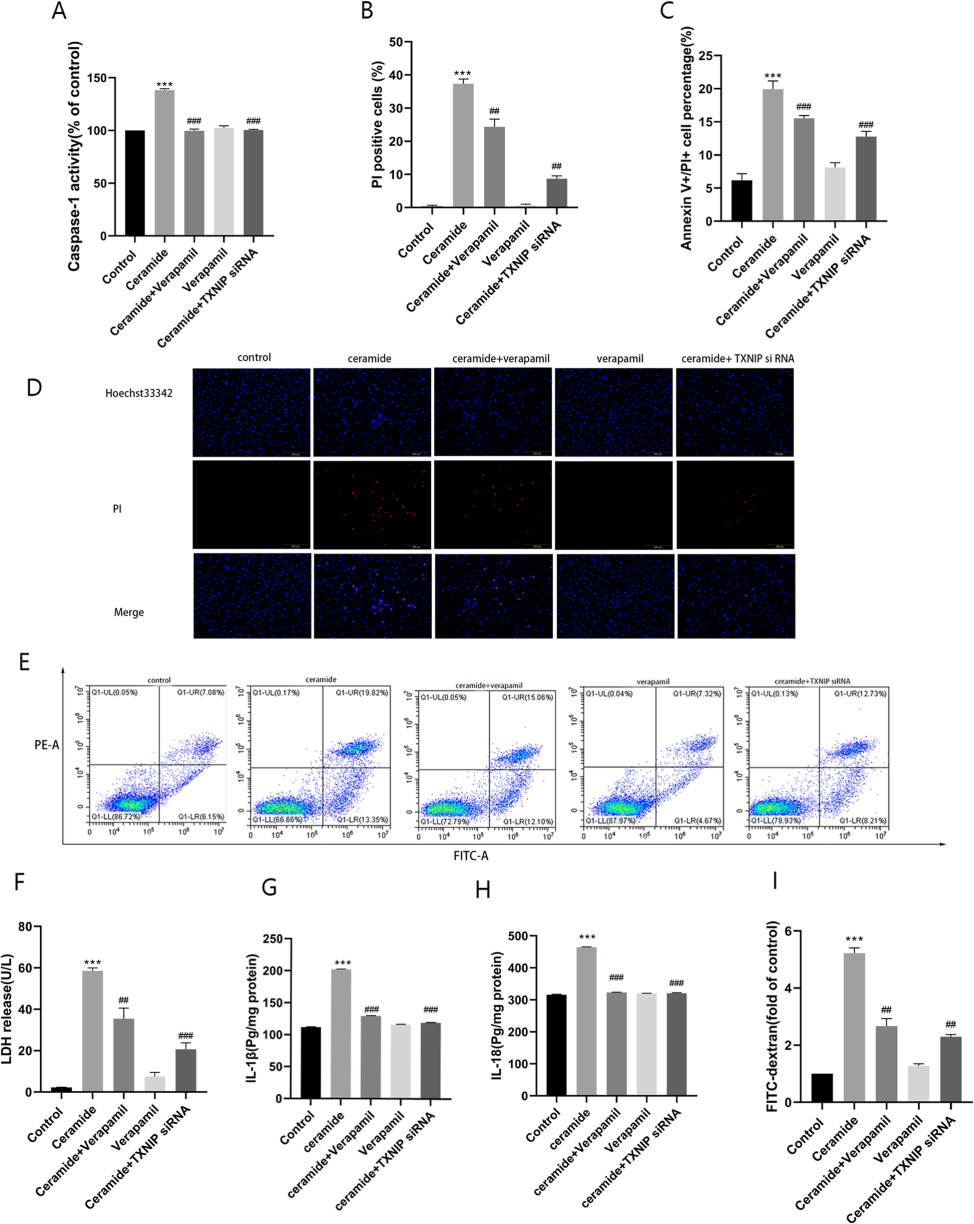
Effects of both pharmacological blockade or downregulation of TXNIP on vascular endothelial injury induced by ceramide. Caspase-1 activity was detected by caspase-1 activity assay kit (**A**). Hoechst33342/PI double staining (**B, D**) and Flow cytometry (**C, E**) were used to evaluate cell death. The cell LDH release was detected by LDH kit (**F**). The levels of IL-1β (**G**) and IL-18 (**H**) in the supernatants were analysed by ELISA. The permeability of endothelial cells was evaluated by FITC-dextran assay (**I**). Values were reported as the mean ± standard deviation in (**A–E**) (*n* = 3) and (**F-I**) (*n* = 5). ^*^P < 0.05, **P < 0.01 and ***P < 0.001 vs control group. ^#^*P* < 0.05, ^##^*P* < 0.01 and ^###^*P* < 0.001 vs ceramide treatment group. Images were captured from random fields of view at a magnification of × 100. Scale bar = 200 μM

We tested our hypothesis on whether TXNIP inhibition alleviates ceramide-induced cell pyroptosis using the small interfering RNA or TXNIP inhibitor (verapamil). Firstly, si-TXNIP and si-NC were transfected into HUVECs by transfection reagent. After verification, mRNA and protein expression levels of TXNIP siRNA group were significantly down-regulated, proving that siRNA effectively silenced TXNIP target genes (Fig. 6A-C). Then, HUVECs were incubated with 20 μM verapamil for 2 h or TXNIP siRNA for 24 h, followed by 20 μM ceramide for 12 h. We found that pre-treatment with verapamil significantly inhibited ceramide-induced levels of pyroptosis-related proteins including NLRP3, TXNIP, caspase-1, casp1 p20, GSDMD, and GSDMD-NT (Fig. 5A-G), and TXNIP siRNA also suppressed expressions of these proteins (Fig. 6D-J). Moreover, pyroptosis was investigated using the caspase-1 activity, PI staining, flow cytometry, LDH release, and ELISA to substantiate this hypothesis. Pre-treatment with verapamil and TXNIP siRNA reduced caspase-1 activity (Fig. 7A), alleviated cell death (Fig. 7B-E), decreased LDH release (Fig. 7F), decreased IL-1β (Fig. 7G) and IL-18 (Fig. 7H) release, and improved ceramide-induced vascular endothelial cell permeability (Fig. 7I) compared to the ceramide group. Therefore, these data suggest that TXNIP upregulation plays a central role in ceramide- induced HUVECs’ pyroptosis.

### Oxidative stress is involved in C8-Cer induced TXNIP/NLRP3/GSDMD pathway in HUVECs

**Fig. 8:**
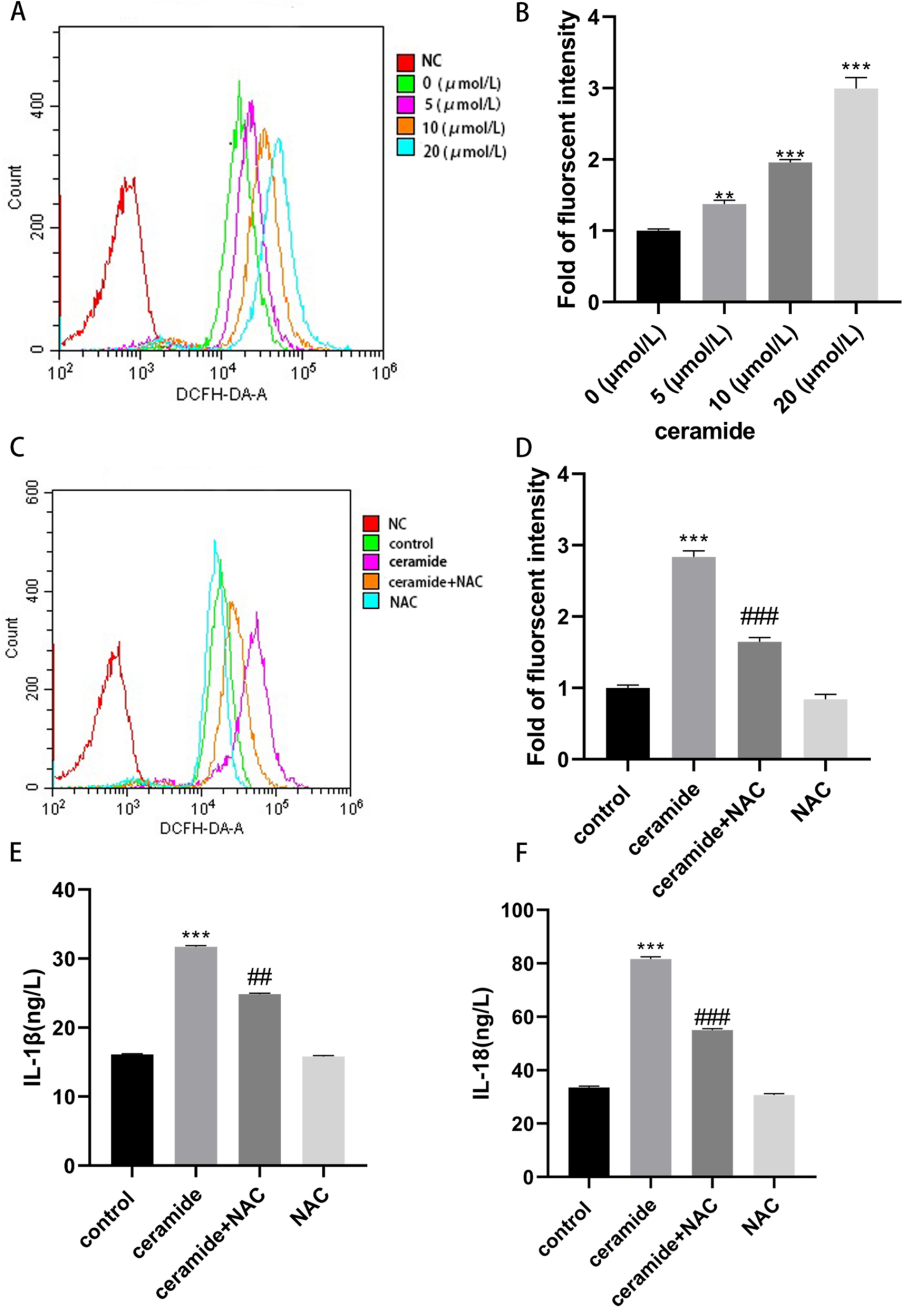
ROS generation mediates ceramide-induced HUVECs’ pyroptosis. HUVECs were treated with various concentrations of C8-ceramide for 12 h, followed by treatment with DCFH-DA for 20 min. **A** The levels of ROS fluorescence were detected by flow cytometry. **B** Measurement of ROS fluorescence intensity using a microplate reader. HUVECs were pre-treated with NAC (10 mM) for 1 h, followed by treatment with 20 μM C8-Cer for 12 h and incubation with DCFH-DA for 20 min. **C** The levels of ROS fluorescence were detected by flow cytometry. **D** Measurement of ROS fluorescence intensity using a microplate reader. **E-F** The levels of IL-1β (**E**) and IL-18 (**F**) in the supernatants were analysed by ELISA. Values were reported as the mean ± standard deviation in (**A–F**) (*n* = 3). ^*^P < 0.05, **P < 0.01 and ***P < 0.001 vs control group. ^#^*P* < 0.05, ^##^*P* < 0.01 and ^###^*P* < 0.001 vs ceramide treatment group

ROS is an efficient molecular signal to trigger pyroptosis. To confirm whether ROS mediated ER stress and mitochondrial dysfunction caused the activation of NLRP3 inflammasome, a ROS inhibitor study was performed. HUVECs were first treated with ceramide at a series of concentrations for 12 h, and then we used a flow cytometer to measure cell fluorescence and a microplate reader to measure the fluorescence intensity of ROS. Results of fluorescence microscopy showed that ceramide significantly increased intracellular ROS level in HUVECs in a concentration dependent manner (Fig. 8A-B). Next, HUVECs were pretreated with the antioxidant N-acetyl-L-cysteine (NAC) (10 mM) for 1 h, followed by treatment with C8-ceramide (20 μM) for 12 h. The ROS scavenger NAC significantly inhibited ceramide-induced ROS generation (Fig. 8C-D) and decreased IL-1β (Fig. 8E) and IL-18 (Fig. 8F) release. These findings support the notion that ceramide induces endothelial cell pyroptosis in a ROS-dependent mechanism.

## Discussion

Ceramides are signalling sphinolipids that play a role not only in cell homeostasis, but also in pathological processes [9]. There is considerable evidence that ceramides have deleterious effects on vascular endothelial function. For example, Ceramides derived from acid sphingomyelinase can lead to increased vascular permeability during sepsis [26]. In lung disease, the upregulation of ceramide has been reported to be involved in the pathogenesis of emphysema by inducing apoptosis of pulmonary vascular endothelial cells [27]. Ceramide also can damage the pulmonary endothelial barrier function by mediating the a nonapoptotic mechanism [28]. Meanwhile, now more and more studies have shown that many diseases are involved in pyroptosis of endothelial cells [29–31]. In view of this, we hypothesised that ceramide is involved in endothelial cell pyroptosis. However, due to the poor solubility of long-chain ceramides, the exogenous transmission of ceramides is limited, so most researchers use short-chain ceramides. At the same time, consider that very short chain ceramides may be non-physiological and/or nonspecific [27]. So we chose C8-ceramide that are more similar to endogenous ceramides. We tested our hypothesis by stimulating HUVECs with C8 ceramide. The results of this in vitro study indicate the important role of C8-ceramide as important mediators in apoptosis, proliferation, and survival [32]. This is the first study to report the pyroptosis effect of ceramides. This may imply that the intervention of pyroptosis in endothelial cells would play a role in the treatment of multiple diseases.

Pyroptosis is a newly discovered form of programmed pro-inflammatory cell death that is distinct from apoptosis and necrosis [33]. Pyroptosis can be divided into classical death pathway that depends on NLRP3 inflammatory vesicles and caspase-1, and the non-classical death pathway that depends on caspase-4 /5/11 [33]. In addition, pyroptosis can be described by pore formation on the membrane due to gasdermin, cell swelling and rapid lysis, and subsequent several pro-inflammatory mediators interleukin-1β (IL-1β) and interleukin-18 (IL-18) release [33–35]. Plenty of studies have demonstrated that pyroptosis is implicated with many diseases, such as atherosclerotic diseases, metabolic diseases, and aseptic inflammatory diseases of important organs [33, 36, 37]. However, it is unclear that the specific mechanism of ceramide-induced endothelial cell pyroptosis in sepsis, acute lung injury and others diseases.

Furthermore, NLRP3 inflammasome-mediated pyroptosis has been identified as the underlying cause of endothelial cell death. It is a polyprotein complex that mediates the activation of caspase-1 [38]. Cleaved caspase-1 promotes the cleavage and translocation of GSDMD, and the gasdermin-N domain accelerates the release of intracellular inflammatory substances by forming a membrane pore [39]. NLRP3 activation is influenced by a variety of cellular proteins or factors, and multiple studies have shown that TXNIP is a significant mediator of NLRP3 inflammasome activation and a target for a variety of diseases, including diabetic nephropathy [40], intestinal ischemia-reperfusion (I/R) injury [41], and sepsis-induced acute kidney injury [42]. It is well-known that TXNIP is a regulator of oxidative stress and participates in the exacerbation of oxidative stress [40, 43]. However, recent studies have shown that TXNIP is also involved in NLRP3 inflammasome-dependent cell pyroptosis [44, 45]. Silencing TXNIP by siRNA or verapamil can prevent TXNIP-NLRP3 binding and subsequent NLRP3 inflammasome activation; moreover, TXNIP inhibition inhibited caspase-1 activity and the production of IL-1β [46]. This suggests that TXNIP/NLRP3 inflammasome function inhibitors may be a promising treatment for endothelial cell pyroptosis. In this study, we found that ceramide facilitates TXNIP expression, upregulates TXNIP-promoted NLRP3 inflammasome formation, and mediates caspase-1 cleavage. More importantly, we found an increase in the N-terminal of GSDMD after HUVECs that were treated with ceramide, indicating the presence of cell pyroptosis. Meanwhile, we observed more PI-positive cell populations and increased LDH, IL-1β, and IL-18 release in ceramide-induced endothelial cells. Unsurprisingly, MCC950 have disrupted the aforementioned positive loop, thereby alleviating vascular endothelial cell damage [47, 48]. Hence, verapamil and TXNIP siRNA ameliorates HUVEC pyroptosis by inhibiting the TXNIP/NLRP3/GSDMD pathway.

Interestingly, ROS affects the activation of the NLRP3 inflammasome by interfering with expression of TXNIP. To be specific, TXNIP binds with TRX in the absence of stimulus, ROS dissociates TXNIP from TRX and facilitates the combining of TXNIP to NLRP3 [15, 49, 50]. In our study, the ROS scavenger NAC significantly inhibited ceramide-induced ROS generation and decreased IL-1βand IL-18 release. These results stated that ROS accumulation induced by ceramide triggers pyroptosis [51].

In summary, this study verified ceramide-induced vascular endothelial injury. Our data also demonstrated that the TXNIP/NLRP3/GSDMD signaling pathway serves a crucial function in ceramide-mediated HUVEC pyroptosis.

## Conclusion

In conclusion, this study found that ceramide-mediated endothelial pyroptosis depends on the activation of ROS-dependent TXNIP/NLRP3/GSDMD signalling, and TXNIP inhibition may provide an important adjuvant therapeutic approach for alleviating vascular endothelial injury in sepsis and other diseases. However, the mechanism of vascular endothelial injury in sepsis and other diseases is intricate, and multiple molecular pathways are involved. This research only describes one of the multiple mechanisms. This study also has limitations. Our experiments were conducted only at the cellular level in vitro and could not be further confirmed by in vivo studies. Furthermore, our study cannot exclude the vital role of apoptosis and necrosis in ceramide-induced vascular endothelial injury.

## Supplementary Information

**Additional file 1: Supplementary original western blot images 1.** Original blots and replicate experimental images corresponding to all the images in Fig. 2. **Supplementary original western blot images 2.** Original blots and replicate experimental images corresponding to all the images in Fig. 5. **Supplementary original western blot images 3.** Original blots and replicate experimental images corresponding to all the images in Fig. 6.

## Data Availability

The data used to support the findings of this study are available from the corresponding author upon request.

## References

[1] Gross CM, Kellner M, Wang T, Lu Q, Sun X, Zemskov EA (2018). LPS-induced acute lung injury involves NF-kappaB-mediated Downregulation of SOX18. Am J Respir Cell Mol Biol.

[2] Zhou R, Huang W, Fan X, Liu F, Luo L, Yuan H (2019). miR-499 released during myocardial infarction causes endothelial injury by targeting alpha7-nAchR. J Cell Mol Med.

[3] Bro-Jeppesen J, Johansson PI, Kjaergaard J, Wanscher M, Ostrowski SR, Bjerre M (2017). Level of systemic inflammation and endothelial injury is associated with cardiovascular dysfunction and vasopressor support in post-cardiac arrest patients. Resuscitation..

[4] Zhou M, Simms HH, Wang P (2004). Adrenomedullin and adrenomedullin binding protein-1 attenuate vascular endothelial cell apoptosis in sepsis. Ann Surg.

[5] Peng F, Chang W, Sun Q, Xu X, Xie J, Qiu H (2020). HGF alleviates septic endothelial injury by inhibiting pyroptosis via the mTOR signalling pathway. Respir Res.

[6] Lai D, Tang J, Chen L, Fan EK, Scott MJ, Li Y (2018). Group 2 innate lymphoid cells protect lung endothelial cells from pyroptosis in sepsis. Cell Death Dis.

[7] Zhang H, Li J, Li L, Liu P, Wei Y, Qian Z (2017). Ceramide enhances COX-2 expression and VSMC contractile hyperreactivity via ER stress signal activation. Vasc Pharmacol.

[8] Jernigan PL, Makley AT, Hoehn RS, Edwards MJ, Pritts TA (2015). The role of sphingolipids in endothelial barrier function. Biol Chem.

[9] Petrache I, Petrusca DN, Bowler RP, Kamocki K (2011). Involvement of ceramide in cell death responses in the pulmonary circulation. Proc Am Thorac Soc.

[10] Gomez-Larrauri A, Presa N, Dominguez-Herrera A, Ouro A, Trueba M, Gomez-Munoz A (2020). Role of bioactive sphingolipids in physiology and pathology. Essays Biochem.

[11] Niaudet C, Bonnaud S, Guillonneau M, Gouard S, Gaugler MH, Dutoit S (2017). Plasma membrane reorganization links acid sphingomyelinase/ceramide to p38 MAPK pathways in endothelial cells apoptosis. Cell Signal.

[12] Wang CY, Xu Y, Wang X, Guo C, Wang T, Wang ZY (2019). Dl-3-n-Butylphthalide inhibits NLRP3 Inflammasome and mitigates Alzheimer's-like pathology via Nrf2-TXNIP-TrX Axis. Antioxid Redox Signal.

[13] Ji Cho M, Yoon SJ, Kim W, Park J, Lee J, Park JG (2019). Oxidative stress-mediated TXNIP loss causes RPE dysfunction. Exp Mol Med.

[14] Jiang L, Fei D, Gong R, Yang W, Yu W, Pan S (2016). CORM-2 inhibits TXNIP/NLRP3 inflammasome pathway in LPS-induced acute lung injury. Inflamm Res.

[15] Xiao YD, Huang YY, Wang HX (2016). Thioredoxin-interacting protein mediates NLRP3 Inflammasome activation involved in the susceptibility to ischemic acute kidney injury in diabetes. Oxidative Med Cell Longev.

[16] Yu Y, Wu DM, Li J, Deng SH, Liu T, Zhang T (2020). Bixin attenuates experimental autoimmune encephalomyelitis by suppressing TXNIP/NLRP3 Inflammasome activity and activating NRF2 signaling. Front Immunol.

[17] Cao Z, Fang Y, Lu Y, Tan D, Du C, Li Y (2017). Melatonin alleviates cadmium-induced liver injury by inhibiting the TXNIP-NLRP3 inflammasome. J Pineal Res.

[18] Zhou R, Tardivel A, Thorens B, Choi I, Tschopp J (2010). Thioredoxin-interacting protein links oxidative stress to inflammasome activation. Nat Immunol.

[19] Jiang J, Shi Y, Cao J, Lu Y, Sun G, Yang J (2021). Role of ASM/Cer/TXNIP signaling module in the NLRP3 inflammasome activation. Lipids Health Dis.

[20] Zhuo L, Chen X, Sun Y, Wang Y, Shi Y, Bu L (2020). Rapamycin inhibited Pyroptosis and reduced the release of IL-1beta and IL-18 in the septic response. Biomed Res Int.

[21] Long Y, Liu X, Tan XZ, Jiang CX, Chen SW, Liang GN (2020). ROS-induced NLRP3 inflammasome priming and activation mediate PCB 118- induced pyroptosis in endothelial cells. Ecotoxicol Environ Saf.

[22] Leng Y, Chen R, Chen R, He S, Shi X, Zhou X (2020). HMGB1 mediates homocysteine-induced endothelial cells pyroptosis via cathepsin V-dependent pathway. Biochem Biophys Res Commun.

[23] Kovacs SB, Miao EA (2017). Gasdermins: effectors of Pyroptosis. Trends Cell Biol.

[24] Zeng C, Duan F, Hu J, Luo B, Huang B, Lou X (2020). NLRP3 inflammasome-mediated pyroptosis contributes to the pathogenesis of non-ischemic dilated cardiomyopathy. Redox Biol.

[25] He Y, Hara H, Nunez G (2016). Mechanism and regulation of NLRP3 Inflammasome activation. Trends Biochem Sci.

[26] Peng H, Li C, Kadow S, Henry BD, Steinmann J, Becker KA (2015). Acid sphingomyelinase inhibition protects mice from lung edema and lethal Staphylococcus aureus sepsis. J Mol Med (Berl).

[27] Medler TR, Petrusca DN, Lee PJ, Hubbard WC, Berdyshev EV, Skirball J (2008). Apoptotic sphingolipid signaling by ceramides in lung endothelial cells. Am J Respir Cell Mol Biol.

[28] Lindner K, Uhlig U, Uhlig S (2005). Ceramide alters endothelial cell permeability by a nonapoptotic mechanism. Br J Pharmacol.

[29] Chen Q, Yang Y, Hou J, Shu Q, Yin Y, Fu W (2019). Increased gene copy number of DEFA1/DEFA3 worsens sepsis by inducing endothelial pyroptosis. Proc Natl Acad Sci U S A.

[30] Mitra S, Exline M, Habyarimana F, Gavrilin MA, Baker PJ, Masters SL (2018). Microparticulate Caspase 1 regulates Gasdermin D and pulmonary vascular endothelial cell injury. Am J Respir Cell Mol Biol.

[31] Wu X, Zhang H, Qi W, Zhang Y, Li J, Li Z (2018). Nicotine promotes atherosclerosis via ROS-NLRP3-mediated endothelial cell pyroptosis. Cell Death Dis.

[32] Yang Y, Xu G, Xu Y, Cheng X, Xu S, Chen S (2021). Ceramide mediates radiation-induced germ cell apoptosis via regulating mitochondria function and MAPK factors in Caenorhabditis elegans. Ecotoxicol Environ Saf.

[33] Coll RC, Schroder K, Pelegrín P (2022). NLRP3 and pyroptosis blockers for treating inflammatory diseases. Trends Pharmacol Sci.

[34] Romero A, Dongil P, Valencia I (2022). Pharmacological blockade of NLRP3 Inflammasome/IL-1β-positive loop mitigates endothelial cell senescence and dysfunction. Aging Dis.

[35] Wang X, Li Q, Sui B, Xu M, Pu Z, Qiu T (2022). Schisandrin a from Schisandra chinensis attenuates Ferroptosis and NLRP3 Inflammasome-mediated Pyroptosis in diabetic nephropathy through mitochondrial damage by AdipoR1 Ubiquitination. Oxidative Med Cell Longev.

[36] Junqueira C, Crespo Â, Ranjbar S (2022). FcγR-mediated SARS-CoV-2 infection of monocytes activates inflammation. Nature..

[37] Tang Y, Yan JH, Ge ZW, Fei AH, Zhang YC (2022). LncRNA Gaplinc promotes the pyroptosis of vascular endothelial cells through SP1 binding to enhance NLRP3 transcription in atherosclerosis. Cell Signal.

[38] Franchi L, Eigenbrod T, Munoz-Planillo R, Nunez G (2009). The inflammasome: a caspase-1-activation platform that regulates immune responses and disease pathogenesis. Nat Immunol.

[39] Yang Y, Liu PY, Bao W, Chen SJ, Wu FS, Zhu PY (2020). Hydrogen inhibits endometrial cancer growth via a ROS/NLRP3/caspase-1/GSDMD-mediated pyroptotic pathway. BMC Cancer.

[40] Han Y, Xu X, Tang C, Gao P, Chen X, Xiong X (2018). Reactive oxygen species promote tubular injury in diabetic nephropathy: the role of the mitochondrial ros-txnip-nlrp3 biological axis. Redox Biol.

[41] Jia Y, Cui R, Wang C, Feng Y, Li Z, Tong Y (2020). Metformin protects against intestinal ischemia-reperfusion injury and cell pyroptosis via TXNIP-NLRP3-GSDMD pathway. Redox Biol.

[42] Li X, Yao L, Zeng X, Hu B, Zhang X, Wang J (2021). miR-30c-5p alleviated Pyroptosis during Sepsis-induced acute kidney injury via targeting TXNIP. Inflammation..

[43] Zhou Y, Chen Z, Yang X, Cao X, Liang Z, Ma H (2021). Morin attenuates pyroptosis of nucleus pulposus cells and ameliorates intervertebral disc degeneration via inhibition of the TXNIP/NLRP3/Caspase-1/IL-1beta signaling pathway. Biochem Biophys Res Commun.

[44] Li N, Zhao T, Cao Y, Zhang H, Peng L, Wang Y (2021). Tangshen formula attenuates diabetic kidney injury by imparting anti-pyroptotic effects via the TXNIP-NLRP3-GSDMD Axis. Front Pharmacol.

[45] An X, Zhang Y, Cao Y, Chen J, Qin H, Yang L (2020). Punicalagin protects diabetic nephropathy by inhibiting Pyroptosis based on TXNIP/NLRP3 pathway. Nutrients..

[46] Pan Z, Shan Q, Gu P, Wang XM, Tai LW, Sun M (2018). miRNA-23a/CXCR4 regulates neuropathic pain via directly targeting TXNIP/NLRP3 inflammasome axis. J Neuroinflammation.

[47] Coll RC, Robertson AA, Chae JJ (2015). A small-molecule inhibitor of the NLRP3 inflammasome for the treatment of inflammatory diseases. Nat Med.

[48] Nalbandian A, Khan AA, Srivastava R (2017). Activation of the NLRP3 Inflammasome is associated with Valosin-containing protein myopathy. Inflammation..

[49] Han CY, Rho HS, Kim A (2018). FXR inhibits endoplasmic reticulum stress-induced NLRP3 Inflammasome in hepatocytes and ameliorates liver injury. Cell Rep.

[50] Koka S, Xia M, Chen Y (2017). Endothelial NLRP3 inflammasome activation and arterial neointima formation associated with acid sphingomyelinase during hypercholesterolemia. Redox Biol.

[51] Mills EL, Kelly B, Logan A (2016). Succinate dehydrogenase supports metabolic repurposing of mitochondria to drive inflammatory macrophages. Cell..

