# Supplementary material for: Ceramide induces pyroptosis through TXNIP/NLRP3/GSDMD pathway in HUVECs
Source: BMC Mol Cell Biol. 2022 Dec 14;23:54. doi: 10.1186/s12860-022-00459-w (PMC9749313; doi:10.1186/s12860-022-00459-w)
Supplement: Supplementary file 1 — Additional file 1: Supplementary original western blot images 1. Original blots and replicate experimental images corresponding to all the images in Fig. 2. Supplementary original western blot images 2. Original blots and replicate experimental images corresponding to all the images in Fig. 5. Supplementary original western blot images 3. Original blots and replicate experimental images corresponding to all the images in Fig. 6. [file 12860_2022_459_MOESM1_ESM.docx]

**Ceramide induces pyroptosis through TXNIP/NLRP3/GSDMD pathway in HUVECs**

Fangfang Liu^1, #^, Yangyang Zhang^1, #^, Yining Shi^2^, Kai Xiong^1^, Fugui Wang^3^, Jin Yang^1, *^

^1^Department of Respiratory and Critical Care Medicine, Second Affiliated Hospital of Anhui Medical University, Hefei, Anhui, China

^2^Department of Respiratory and Critical Care Medicine, Chest Hospital of Anhui Province, Hefei, Anhui, China

^3^Department of Emergency Medicine, Second Affiliated Hospital of Anhui Medical University, Hefei, Anhui, China

^*^Correspondence author at: Department of Respiratory and Critical Care Medicine, Second Affiliated Hospital of Anhui Medical University, Hefei, Anhui, 230601, China; and Institute of Respiratory Diseases，Second Affiliated Hospital of Anhui Medical University, Hefei, Anhui, 230601, China.

E-mail addresses: yangqj1015@foxmail.com

^#^Contributed equally

NLRP3


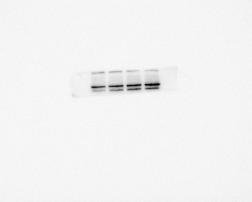


120KD


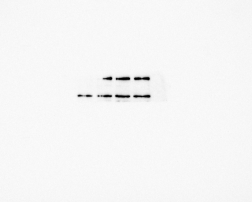


120KD


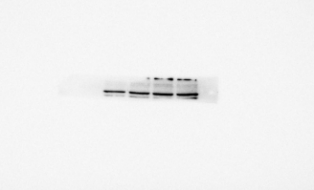


120KD

TXNIP


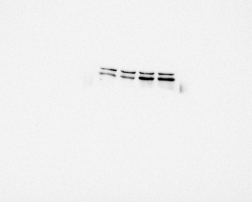


60KD

50KD

40KD


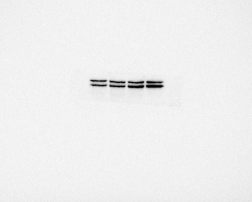


60KD

50KD

40KD


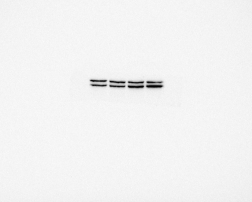


60KD

50KD

40KD

GSDMD


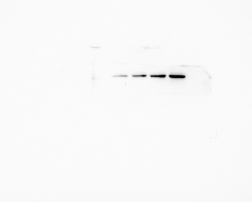


60KD

50KD


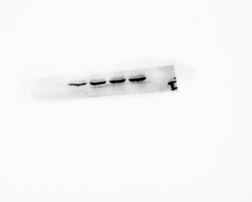


60KD

50KD


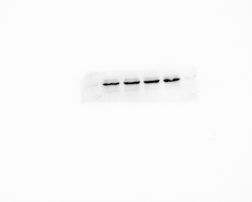


60KD

50KD

NT


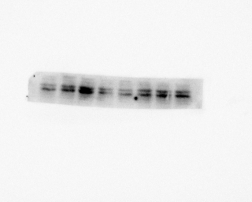


30KD

40KD


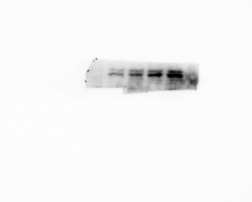


30KD

40KD


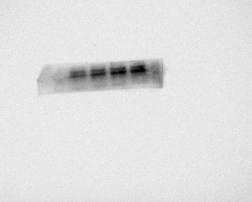


30KD

40KD

Caspase-1


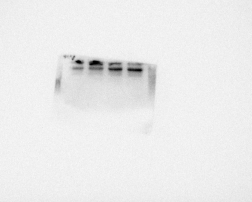


40KD

30KD


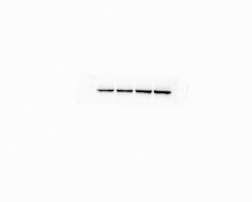


40KD

30KD


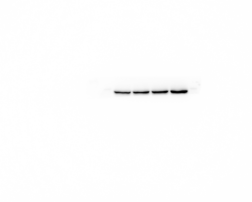


40KD

30KD

p20

20KD


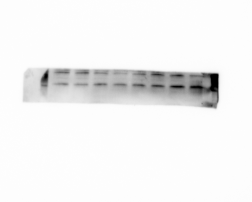


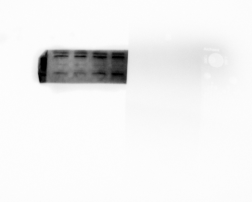


20KD


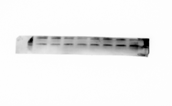


20KD

Actin


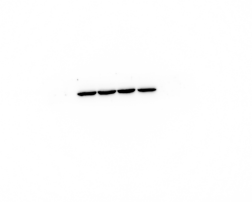


40KD

50KD


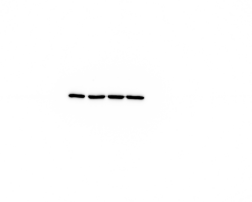


40KD

50KD


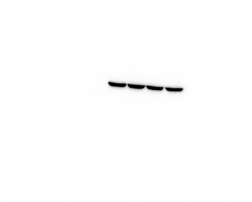


40KD

50KD

**Supplementary original western blot images 1. Original blots and replicate experimental images corresponding to all the images in Fig. 2.**

All Western blot images were showed. We outlined the edges of the blots using solid deep color lines, and the valid regions of the original blots related to main figures were denoted using red boxes. The membrane was tailored according to the molecular weight of the target protein before incubated with primary antibodies.

NLRP3


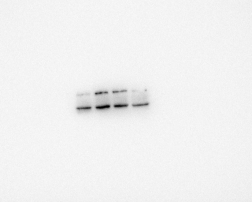


120KD


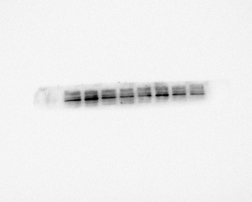


120KD

TXNIP


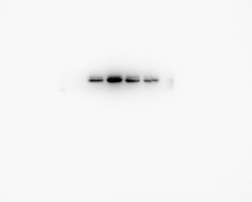


60KD

50KD

40KD


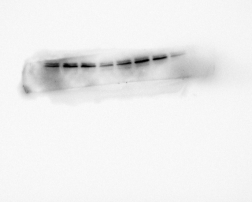


60KD

50KD

40KD

GSDMD


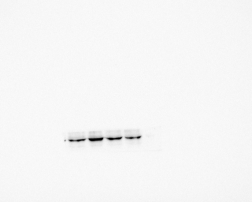


60KD

50KD


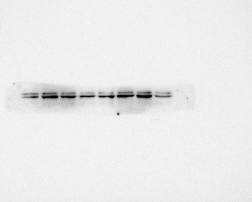


60KD

50KD


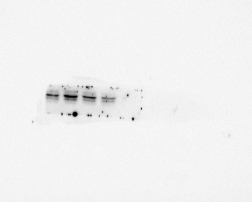


60KD

50KD

NT


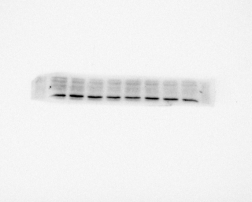


30KD

40KD


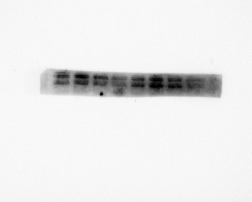


30KD

40KD

caspase-1


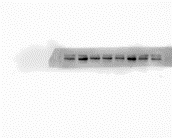


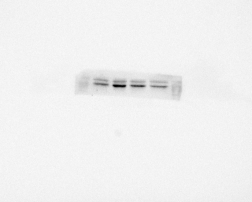


40KD

30KD

40KD

30KD

p20


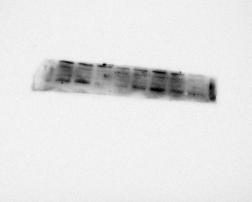


20KD


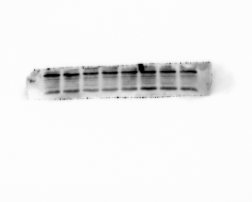


20KD

Actin


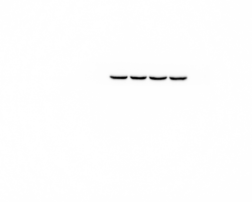


40KD

50KD


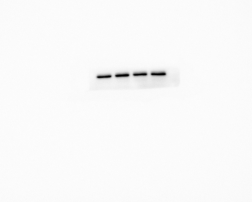


40KD

50KD


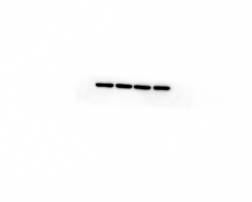


40KD

50KD

**Supplementary original western blot images 2. Original blots and replicate experimental images corresponding to all the images in Fig. 5.**

All Western blot images were showed. We outlined the edges of the blots using solid deep color lines, and the valid regions of the original blots related to main figures were denoted using red boxes. The membrane was tailored according to the molecular weight of the target protein before incubated with primary antibodies.

TXNIP


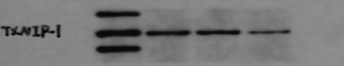


60KD

50KD

40KD


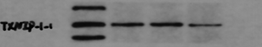


60KD

50KD

40KD


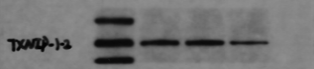


60KD

50KD

40KD

Actin


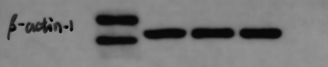


40KD

50KD


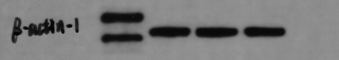


40KD

50KD


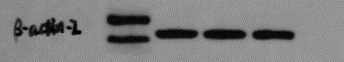


40KD

50KD

TXNIP


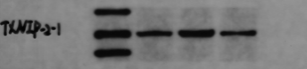


60KD

50KD

40KD


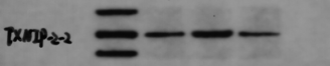


60KD

50KD

40KD


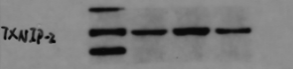


60KD

50KD

40KD

NLRP3


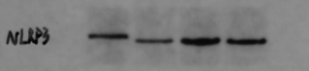


120KD


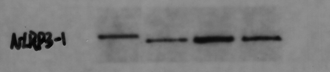


120KD


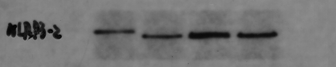


120KD

GSDMD


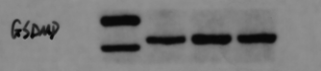


60KD

50KD


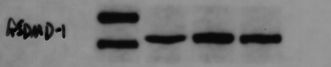


60KD

50KD


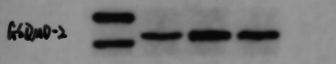


60KD

50KD

NT


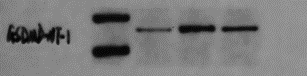


30KD

40KD


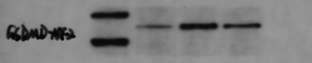


30KD

40KD


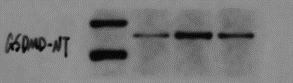


30KD

40KD


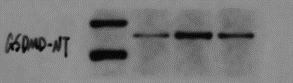


caspase-1 / P20


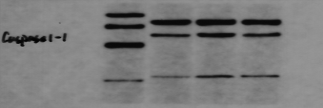


20KD

40KD

30KD


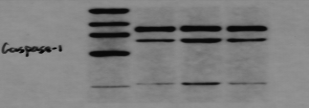


20KD

40KD

30KD

40KD

30KD


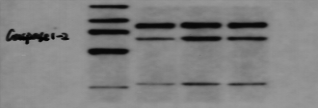


20KD

Actin


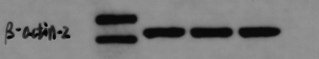


40KD

50KD


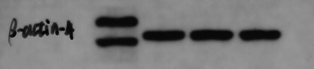


40KD

50KD


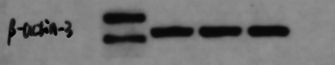


40KD

50KD

**Supplementary original western blot images 3. Original blots and replicate experimental images corresponding to all the images in Fig. 6.**

All Western blot images were showed. We outlined the edges of the blots using solid deep color lines. The membrane was tailored according to the molecular weight of the target protein before incubated with primary antibodies.
